# Impact of Preoperative and Intraoperative Factors on Postoperative Outcomes in Patients with Colorectal Cancer: A 10-Year Retrospective Study

**DOI:** 10.3390/diseases13010016

**Published:** 2025-01-15

**Authors:** Lucian Flavius Herlo, Ioana Golu, Alexandra Herlo, Claudia Raluca Balasa Virzob, Ionescu Alin, Stela Iurciuc, Ionut Eduard Iordache, Luana Alexandrescu, Doina Ecaterina Tofolean, Raluca Dumache

**Affiliations:** 1 Doctoral School, “Victor Babes” University of Medicine and Pharmacy Timisoara, Eftimie Murgu Square 2, 300041 Timisoara, Romania; flavius.herlo@umft.ro; 2 Department of Internal Medicine II, “Victor Babes” University of Medicine and Pharmacy Timisoara, Eftimie Murgu Square 2, 300041 Timisoara, Romania; 3 University Clinic of Endocrinology, “Victor Babes” University of Medicine and Pharmacy Timisoara, Eftimie Murgu Square 2, 300041 Timisoara, Romania; 4 Center for Molecular Research in Nephrology and Vascular Disease, “Victor Babes” University of Medicine and Pharmacy Timisoara, Eftimie Murgu Square 2, 300041 Timisoara, Romania; 5 Department XIII, Discipline of Infectious Diseases, “Victor Babes” University of Medicine and Pharmacy Timisoara, 2 Eftimie Murgu Square, 300041 Timisoara, Romania; alexandra.mocanu@umft.ro; 6 Department of Clinic Nursing, “Victor Babes” University of Medicine and Pharmacy Timisoara, 2 Eftimie Murgu Square, 300041 Timisoara, Romania; virzob.claudia@umft.ro; 7 Department XVI, Discipline of Family Medicine, “Victor Babes” University of Medicine and Pharmacy Timisoara, Eftimie Murgu Square 2, 300041 Timisoara, Romania; ionescu.alin@umft.ro; 8 Cardiology Department, “Victor Babes” University of Medicine and Pharmacy Timisoara, Eftimie Murgu Square 2, 300041 Timisoara, Romania; iurciuc.stela@umft.ro; 9 Department of General Surgery, Faculty of Medicine and Pharmacy Constanta, Ovidius University, 900470 Constanta, Romania; eduardiordache@yahoo.com; 10 Department of Gastroenterology, Faculty of Medicine and Pharmacy Constanta, Ovidius University, 900470 Constanta, Romania; alexandrescu_l@yahoo.com (L.A.); tofoleandoina@yahoo.com (D.E.T.); 11 Department of Forensic Medicine, Bioethics, Medical Ethics and Medical Law, “Victor Babes” University of Medicine and Pharmacy Timisoara, Eftimie Murgu Square 2, 300041 Timisoara, Romania; raluca.dumache@umft.ro; 12 Center for Ethics in Human Genetic Identifications, “Victor Babes” University of Medicine and Pharmacy Timisoara, Eftimie Murgu Square 2, 300041 Timisoara, Romania

**Keywords:** colorectal cancer, postoperative complications, preoperative factors, intraoperative factors, surgical outcomes, CRP, albumin, blood loss

## Abstract

Background and Objectives: Colorectal cancer is a major contributor to global cancer morbidity and mortality. Surgical resection remains the cornerstone of treatment, but postoperative complications can significantly affect patient outcomes. Identifying factors that influence postoperative morbidity and mortality is crucial for optimizing patient care. This study aims to evaluate the impact of preoperative, intraoperative, and postoperative factors on surgical outcomes in patients with colorectal cancer. Methods: A retrospective cohort study was conducted on 688 patients who underwent colorectal cancer surgery within a 10-year period. Data collected included demographic information, comorbidities, laboratory values, surgical details, and postoperative outcomes. Statistical analyses were performed using chi-square tests for categorical variables and t-tests for continuous variables. Multivariate logistic regression was used to identify independent predictors of postoperative complications and mortality. Results: Postoperative complications occurred in 28.5% of patients, and the 30-day mortality rate was 5.2%. Preoperative factors such as elevated C-reactive protein (CRP) levels (*p* < 0.001), low albumin levels (*p* = 0.003), a high American Society of Anesthesiologists (ASA) score (*p* < 0.001), and presence of comorbidities like diabetes and hypertension (*p* = 0.005) were significantly associated with increased postoperative complications. Intraoperative factors such as blood loss greater than 500 mL (*p* < 0.001) and longer operative time (*p* = 0.021) were also significant predictors of adverse outcomes. Multivariate analysis identified elevated CRP (OR 2.1, 95% CI 1.5–2.9), low albumin (OR 1.8, 95% CI 1.3–2.5), and blood loss > 500 mL (OR 2.4, 95% CI 1.7–3.4) as independent predictors of postoperative complications. Conclusions: Preoperative inflammatory markers, nutritional status, ASA score, comorbidities, and intraoperative factors like blood loss significantly influence postoperative outcomes in colorectal cancer surgery. Recognizing these risk factors allows for better preoperative optimization and surgical planning, potentially reducing postoperative morbidity and mortality.

## 1. Introduction

Colorectal cancer is the third most common cancer worldwide and the second leading cause of cancer-related deaths, accounting for approximately 10% of all cancer incidences and mortalities globally [1]. Surgical resection remains the primary treatment modality for localized colorectal cancer, offering the best chance for a cure [2]. Despite advances in surgical techniques and perioperative care, postoperative complications continue to pose significant challenges, affecting patient recovery, hospital stay, and overall survival [3].

Postoperative complications not only increase morbidity and mortality but also lead to prolonged hospitalization and higher healthcare costs [4]. Identifying patients at higher risk of complications allows clinicians to implement targeted interventions to mitigate these risks [5]. Various factors, including patient demographics, comorbidities, nutritional status, and intraoperative variables, have been studied for their impact on surgical outcomes [6].

Inflammatory markers such as C-reactive protein (CRP) and albumin levels have emerged as potential predictors of postoperative complications [7]. Elevated CRP levels indicate systemic inflammation, which has been associated with poor surgical outcomes [8]. Hypoalbuminemia reflects malnutrition and has been linked to impaired wound healing and increased susceptibility to infections [9]. Additionally, the American Society of Anesthesiologists (ASA) score is a widely used preoperative assessment tool to evaluate patients’ overall health status and predict perioperative risks [10].

Intraoperative factors like blood loss and operative time are also crucial determinants of postoperative outcomes [11]. Excessive blood loss can lead to hemodynamic instability and transfusion-related complications [12]. Prolonged operative time may increase the risk of infections due to extended exposure and tissue handling [13]. Despite the recognition of these factors, there is a need for comprehensive studies that simultaneously evaluate the impact of multiple preoperative and intraoperative variables on postoperative outcomes in colorectal cancer surgery.

This study aims to bridge this gap by conducting a retrospective analysis of 688 patients undergoing colorectal cancer surgery. By examining a wide range of variables, including laboratory values, comorbidities, and surgical details, we seek to identify significant predictors of postoperative complications and mortality. Understanding these associations can inform clinical practice, allowing for better risk stratification and personalized patient care.

## 2. Materials and Methods

### 2.1. Study Design, Population, and Ethical Considerations

This retrospective cohort study was conducted at the Department of Surgery affiliated with the Victor Babes University of Medicine and Pharmacy Timisoara, Romania, analyzing data from 688 patients who underwent surgical resection for colorectal cancer between January 2014 and December 2023. Inclusion criteria were patients aged 18 years and older with a confirmed diagnosis of colorectal adenocarcinoma who underwent elective surgery. Exclusion criteria included emergency surgeries, patients with metastatic disease requiring palliative procedures, and those with incomplete medical records.

Patients were identified through the hospital’s surgical database. Demographic data, comorbidities, laboratory results, surgical details, and postoperative outcomes were extracted from electronic medical records. The study was approved by the Institutional Review Board, and patient confidentiality was maintained throughout the research process. Ethical approval was secured from the Institutional Review Boards of the hospital, with the approval number E-1305/27 February 2024. The study was conducted in strict adherence to the ethical principles outlined in the Declaration of Helsinki.

### 2.2. Data Collection and Variables

Data collected included patient demographics (age, sex), lifestyle factors (smoking status, alcohol consumption), comorbidities (diabetes, hypertension, chronic obstructive pulmonary disease), and preoperative laboratory values (CRP, albumin, hemoglobin, white blood cell count). Nutritional status was assessed using body mass index (BMI) and serum albumin levels. Surgical details recorded were type of surgery (laparoscopic vs. open), operative time, intraoperative blood loss, and intraoperative complications. Postoperative outcomes included length of hospital stay, postoperative complications (classified according to the Clavien–Dindo classification), and 30-day mortality. Laboratory values were measured using standard hospital protocols. CRP levels were categorized as normal (<10 mg/L) or elevated (≥10 mg/L). Albumin levels were considered low if <35 g/L. The ASA score was assigned preoperatively by the anesthesiologist.

### 2.3. Statistical Analysis

Statistical analyses were performed using SPSS version 25.0 (IBM Corp., Armonk, NY, USA). Continuous variables were expressed as mean ± standard deviation or median (interquartile range) based on data distribution. Categorical variables were presented as frequencies and percentages. Comparisons between groups (patients with and without postoperative complications) were made using the chi-square test or Fisher’s exact test for categorical variables and the independent *t*-test or Mann–Whitney U test for continuous variables, as appropriate. Variables with a *p*-value < 0.05 in univariate analysis were included in a multivariate logistic regression model to identify independent predictors of postoperative complications. Odds ratios (OR) with 95% confidence intervals (CI) were calculated. A *p*-value < 0.05 was considered statistically significant.

## 3. Results

Table 1 summarizes the demographic and clinical characteristics of the 688 patients included in the study, divided into those who experienced postoperative complications (n = 196) and those who did not (n = 492). The mean age of patients with complications was significantly higher (68 ± 11 years) compared to those without complications (64 ± 12 years), with a *p*-value of <0.001, indicating that older age is associated with increased risk of complications. There was no significant difference in sex distribution between the groups (*p* = 0.712), suggesting that gender did not influence complication rates.

BMI was slightly lower in the complication group (25.8 ± 4.5 kg/m^2^) compared to the no complication group (26.8 ± 4.0 kg/m^2^), with a *p*-value of 0.005, indicating a potential association between lower BMI and higher complication rates. Lifestyle factors such as smoking and alcohol consumption were more prevalent in the complication group, with smoking rates at 36.7% vs. 28.0% (*p* = 0.023) and alcohol consumption at 29.6% vs. 21.5% (*p* = 0.021). This suggests that these factors may contribute to poorer postoperative outcomes.

Comorbidities like diabetes mellitus and hypertension were significantly more common in patients who experienced complications. Diabetes was present in 29.6% of patients with complications compared to 19.1% without (*p* = 0.003), and hypertension was observed in 52.0% vs. 42.7% (*p* = 0.027). A higher ASA score (≥3) was markedly more frequent in the complication group (63.3% vs. 32.5%, *p* < 0.001), emphasizing its role as a predictor of postoperative risk.

Table 2 presents the preoperative laboratory values of patients, comparing those with and without postoperative complications. CRP levels were significantly higher in the complication group, with a median of 18 mg/L compared to 10 mg/L in the no-complication group (*p* < 0.001). Elevated CRP indicates systemic inflammation, which may predispose patients to postoperative complications.

Serum albumin levels were significantly lower in patients who developed complications (35 ± 6 g/L) versus those who did not (39 ± 4 g/L), with a *p*-value of <0.001. Hypoalbuminemia reflects poor nutritional status and is associated with impaired healing and increased susceptibility to infections. Hemoglobin levels were also lower in the complication group (11.8 ± 2.0 g/dL vs. 12.8 ± 1.6 g/dL, *p* < 0.001), suggesting that anemia may contribute to adverse outcomes.

White blood cell (WBC) counts were higher in patients with complications (8.2 ± 2.5 × 10^9^/L) compared to those without (7.2 ± 1.9 × 10^9^/L, *p* < 0.001), indicating a possible ongoing infection or inflammatory response. Platelet counts were slightly higher in the complication group (265 ± 80 × 10^9^/L vs. 245 ± 65 × 10^9^/L, *p* = 0.002), which may be related to inflammation or a reactive process. 

Table 3 details the surgical variables associated with the patients’ procedures. Laparoscopic surgery was performed in 45.3% of cases but was less common in the complication group (34.7%) compared to the no-complication group (49.6%), with a significant *p*-value of <0.001. This suggests that laparoscopic surgery may be associated with fewer postoperative complications, potentially due to its minimally invasive nature.

Operative time was longer in patients who developed complications (195 ± 50 min) versus those who did not (175 ± 40 min), with a *p*-value of <0.001. Longer surgeries may increase the risk of complications due to prolonged anesthesia and greater tissue exposure. Blood loss greater than 500 mL occurred in 38.8% of patients with complications compared to 10.6% without complications (*p* < 0.001), highlighting significant blood loss as a predictor of adverse outcomes.

Intraoperative complications were significantly higher in the complication group (18.4% vs. 4.9%, *p* < 0.001). The types of resections performed did not differ significantly between the groups (*p* > 0.05), indicating that the location of the tumor and type of surgical resection were not associated with postoperative complications in this cohort.

Table 4 presents postoperative outcomes. Patients with complications had a significantly longer hospital stay (14 ± 5 days) compared to those without complications (8 ± 3 days), with a *p*-value of <0.001. The reoperation rate was markedly higher in the complication group (16.3% vs. 1.6%, *p* < 0.001), indicating that complications often necessitated additional surgical interventions.

The 30-day mortality rate was significantly higher among patients who experienced complications (14.3%) compared to those who did not (1.6%), with a *p*-value of <0.001. This underscores the impact of postoperative complications on patient survival. The severity of complications was assessed using the Clavien–Dindo classification, with Grades I–II representing minor complications and Grades III–IV representing major complications.

In patients younger than 65, those with elevated CRP levels have a 43.6% complication rate, significantly higher than the 18.7% in those with normal CRP. For patients aged 65 and above, the complication rate jumps to 67.9% for elevated CRP, compared to 31.2% with normal CRP. In surgical type subgroups, laparoscopic surgery shows a complication rate of 28.4% with elevated CRP versus 15.8% with normal CPR, while open surgery displays a more pronounced disparity—62.3% versus 34.1%. BMI subgroups also show variation, with higher complications at 39.7% for elevated CRP in patients with a BMI ≥ 25 kg/m^2^, compared to 21.9% with normal CRP; and 54.6% versus 26.8% in those with a BMI < 25 kg/m^2^ (Table 5).

Table 6 displays the univariate analysis of factors associated with postoperative complications. Age over 65 years was associated with an increased risk of complications (OR 1.8, *p* < 0.001). Low BMI (<18.5 kg/m^2^) also increased the risk (OR 1.5, *p* = 0.012), suggesting that underweight patients are more susceptible to adverse outcomes.

Lifestyle factors such as smoking and alcohol consumption were significant predictors, both with an OR of 1.5 (*p* < 0.05). Comorbidities like diabetes mellitus (OR 1.8, *p* < 0.001) and hypertension (OR 1.4, *p* = 0.036) were associated with higher complication rates. An ASA score of ≥3 significantly increased the risk (OR 3.5, *p* < 0.001), reinforcing its value in preoperative risk assessment.

Elevated CRP and low albumin levels were strong predictors of complications, with ORs of 2.3 and 2.0, respectively (*p* < 0.001). Anemia (hemoglobin < 10 g/dL) and elevated WBC counts were also significant factors. Not undergoing laparoscopic surgery increased the risk (OR 1.9, *p* < 0.001), as did operative time over 180 min (OR 1.6, *p* = 0.002). The most substantial risk was associated with blood loss greater than 500 mL (OR 5.3, *p* < 0.001) and intraoperative complications (OR 4.4, *p* < 0.001).

In the study, several variables identified in the univariate analysis as significant predictors of postoperative complications did not make it into the multivariate logistic regression model, including BMI, smoking, alcohol consumption, diabetes mellitus, hypertension, hemoglobin levels, WBC count, lack of laparoscopic surgery, and operative time. The multivariate analysis was adjusted for confounders by including only variables that maintained their significance when controlling for others, focusing on age over 65, ASA score ≥ 3, elevated CRP, low albumin, significant blood loss (>500 mL), and intraoperative complications to evaluate their independent impact on postoperative outcomes.

Table 7 presents the multivariate logistic regression analysis identifying independent predictors of postoperative complications. Age over 65 years remained a significant predictor (adjusted OR 1.5, *p* = 0.021). An ASA score of ≥3 was strongly associated with complications (adjusted OR 2.8, *p* < 0.001), emphasizing its importance in preoperative evaluation. Elevated CRP levels independently predicted complications (adjusted OR 2.1, *p* < 0.001), indicating that systemic inflammation is a significant risk factor. Low albumin levels also remained significant (adjusted OR 1.8, *p* < 0.001), highlighting the role of nutritional status. Blood loss greater than 500 mL was a strong independent predictor (adjusted OR 2.4, *p* < 0.001), suggesting that minimizing intraoperative blood loss could improve outcomes. Intraoperative complications increased the risk of postoperative complications nearly threefold (adjusted OR 2.9, *p* < 0.001), underscoring the impact of surgical events on patient recovery.

## 4. Discussion

### 4.1. Literature Findings

The present study evaluated 688 patients undergoing colorectal cancer surgery to identify factors associated with postoperative complications and mortality. Our findings indicate that both preoperative and intraoperative factors significantly influence postoperative outcomes. Older age was associated with higher complication rates, consistent with previous studies indicating that aging is linked to reduced physiological reserve and increased vulnerability to surgical stress [14]. An ASA score of ≥3 emerged as a strong predictor of complications, reinforcing its utility in assessing perioperative risk [15].

Elevated preoperative CRP levels were independently associated with postoperative complications. CRP, a marker of systemic inflammation, has been linked to poorer surgical outcomes in colorectal cancer [16]. Similarly, low albumin levels, indicative of malnutrition, were significant predictors. Nutritional deficits can impair wound healing and immune function, increasing susceptibility to infections [17]. Many pathogenic bacteria produce CRC-causing toxins. Genotoxins, virulence factors, gut microbial metabolites, inflammation pathways, oxidative stress, and anti-oxidative defense regulation are the main ways gut microbiota cause colorectal cancer. ETBF produces [18] B. fragilis toxin, which activates NF-κB and Wnt/β-catenin pathways, causing cell proliferation, DNA damage, and pro-inflammatory mediator release [19]. Although toxin-producing bacteria are a tiny part of the gut microbiota, CRC tissue samples show high toxin expression. Thus, addressing these poisons may treat CRC [20].

Intraoperative factors such as significant blood loss and intraoperative complications were strong independent predictors of adverse outcomes. Excessive blood loss can lead to hemodynamic instability and necessitate transfusions, which are associated with immunomodulation and infection risk [21]. Intraoperative complications reflect surgical difficulty and may prolong operative time, further increasing risk [22].

The lower rate of complications in patients undergoing laparoscopic surgery suggests benefits of minimally invasive techniques. Laparoscopic procedures are associated with reduced tissue trauma, lower infection rates, and quicker recovery [23]. However, patient selection biases and surgeon expertise may influence these outcomes.

In their retrospective study, Young Wan Kim and Ik Yong Kim [24] analyzed factors contributing to postoperative complications and one-year mortality in 204 octogenarians and nonagenarians undergoing colorectal surgery, finding a 26% complication rate and 2% 30-day mortality. Significant predictors included older age (≥90 years), higher ASA scores (≥3), combined surgery, and lower levels of hemoglobin and albumin. Similarly, Toru Aoyama et al. [25], using data from three large phase-III trials with 5530 patients, demonstrated that postoperative complications significantly reduced five-year overall and disease-free survival rates (68.9% and 74.8% for patients with complications vs. 75.8% and 82.2% for those without). Both studies highlight the impact of complications on mortality and long-term survival in colorectal cancer patients, emphasizing the importance of tailored surgical approaches and vigilant postoperative care to improve outcomes, particularly in older adults.

In their retrospective analysis, Toshinori Sueda et al. [26] assessed the prognostic impact of postoperative intra-abdominal infections on stage I–III colorectal cancer patients, determining that such infections significantly shortened local recurrence-free survival. By utilizing a propensity score-matched analysis to compare 62 patients from a total cohort of 755, the study found a clear link between these infections and increased local recurrence (*p* = 0.05 after matching), though it did not affect other survival metrics significantly. In a similar manner, the study by Hiroya Matsuoka et al. [27] focused on stage III colorectal cancer patients and identified the postoperative C-reactive protein/albumin ratio (CAR) as an independent predictor of recurrence-free and overall survival. Their research on 133 patients highlighted that a higher postoperative CAR significantly correlated with poorer outcomes, suggesting its utility as a biomarker for assessing the need for adjuvant chemotherapy.

Chang Kyu Oh et al. [28] investigated the impact of postoperative complications on long-term oncologic outcomes in 310 patients undergoing radical colorectal cancer surgery, categorizing complications into minor and major based on the extended Clavien–Dindo classification. They found no significant difference in 5-year disease-free survival between the minor complication group (84.4%) and the major complication group (78.5%), suggesting that the severity of complications post-surgery did not impact the long-term disease-free survival of colorectal cancer patients. In a similar manner, Amal A Alzahrani et al. [29] conducted a study at King Abdulaziz University Hospital, examining 195 patients who underwent colorectal cancer surgeries and documented a 29.7% rate of postoperative complications. Their findings highlight that certain preoperative and intraoperative factors, such as low albumin levels, high white blood cell count, and higher American Society of Anesthesiologists (ASA) scores, significantly increased the likelihood of intraoperative complications. Both studies emphasize the complexity of managing colorectal cancer postoperatively, indicating that while some factors like surgical complications and physiological stress markers impact immediate postoperative outcomes, they do not necessarily predict long-term oncologic results, underscoring the need for tailored patient management strategies to optimize both immediate and long-term outcomes.

Michael Osseis and colleagues analyzed the impact of postoperative complications (POCs) on the long-term survival of patients who underwent surgery for T4 colorectal cancer at a single center from 2004 to 2013 [30]. The study included 106 patients, 46 of whom (43%) developed POCs, with 9 experiencing severe complications (Clavien–Dindo ≥ grade III). However, the presence of POCs did not significantly affect overall survival (OS) or recurrence-free survival (RFS), with OS rates of 65% for patients with POCs versus 69% without, and RFS rates of 58% versus 70%, respectively. In a similar manner, the study conducted by Edgar Ernesto Vergara Dagobeth et al. [6] in a Colombian Caribbean population also focused on postoperative complications in colorectal cancer surgery, including 84 patients and identifying factors such as the anatomical subsite of the neoplasm, intraoperative complications, and intensive care stays as associated with postoperative outcomes. Unlike Osseis et al., Vergara Dagobeth’s study found specific intraoperative and care-related factors that significantly influenced surgical outcomes, suggesting regional variations in the impacts of complications on oncological results.

Nevertheless, in future studies on the impact of preoperative and intraoperative factors on postoperative outcomes in colorectal cancer, incorporating additional parameters such as pre-operative sarcopenia and hypophosphatemia could substantially refine risk stratification. Sarcopenia, characterized by the degenerative loss of skeletal muscle mass and strength, has been linked to increased surgical risks and poorer outcomes, particularly in oncological surgeries [31]. It affects the patient’s ability to recover from major surgery due to diminished physiological reserves and is associated with longer hospital stays and higher rates of complications and mortality. Similarly, hypophosphatemia, indicative of poor nutritional status, can impair energy metabolism and cellular function, crucial for postoperative recovery and wound healing [32]. Integrating these parameters would likely enhance the predictive accuracy of predictive models, providing a more comprehensive evaluation of a patient’s readiness for surgery and potential for recovery.

Our study underscores the importance of comprehensive preoperative assessment and optimization. Addressing modifiable risk factors such as nutritional deficiencies and systemic inflammation may improve outcomes. Intraoperative strategies to minimize blood loss and prevent complications are also crucial.

### 4.2. Study Limitations and Future Perspectives

This study has several limitations. Firstly, its retrospective design may introduce selection bias and limit the ability to establish causality. While we adjusted for confounding factors in multivariate analysis, unmeasured variables may still influence the results. Secondly, data were collected from a single institution, which may limit the generalizability of the findings to other settings with different patient populations or surgical practices.

Thirdly, some variables, such as smoking and alcohol consumption, relied on patient self-reporting, which may be subject to reporting bias. Additionally, we did not assess postoperative functional outcomes or quality of life measures, which are important considerations in evaluating the full impact of surgical interventions.

Finally, the study period spans several years, during which advancements in surgical techniques and perioperative care may have occurred. While this reflects real-world practice, it may also introduce variability in patient management that could affect outcomes. Moreover, the study only provides early postoperative outcomes, while long-term follow-up would better reflect the impact of the studied variables. Future prospective, multicenter studies are needed to validate these findings and explore interventions to mitigate identified risk factors.

## 5. Conclusions

In conclusion, our study identified several key preoperative and intraoperative factors associated with increased postoperative complications in colorectal cancer surgery. Elevated CRP levels and low albumin indicate that systemic inflammation and poor nutritional status significantly impact surgical outcomes. An ASA score of ≥3 highlights the importance of overall patient health in predicting risk. Intraoperative factors, particularly excessive blood loss and intraoperative complications, further contribute to adverse outcomes.

## Figures and Tables

**Table 1:** Demographic and clinical characteristics of patients.

Variable	Total (n = 688)	Complications (n = 196)	No Complications (n = 492)	*p*-Value
Age (Years)	65 ± 12	68 ± 11	64 ± 12	<0.001
Sex (Male/Female)	384/304	110/86	274/218	0.712
BMI (kg/m^2^)	26.5 ± 4.2	25.8 ± 4.5	26.8 ± 4.0	0.005
Smoking (%)	210 (30.5%)	72 (36.7%)	138 (28.0%)	0.023
Alcohol Consumption (%)	164 (23.8%)	58 (29.6%)	106 (21.5%)	0.021
Diabetes Mellitus (%)	152 (22.1%)	58 (29.6%)	94 (19.1%)	0.003
Hypertension (%)	312 (45.3%)	102 (52.0%)	210 (42.7%)	0.027
ASA Score (≥3) (%)	284 (41.3%)	124 (63.3%)	160 (32.5%)	<0.001

**Table 2:** Preoperative laboratory values.

Laboratory Parameter	Total (n = 688)	Complications (n = 196)	No Complications (n = 492)	*p*-Value
CRP (mg/L)	12 (5–25)	18 (10–35)	10 (4–20)	<0.001
Albumin (g/L)	38 ± 5	35 ± 6	39 ± 4	<0.001
Hemoglobin (g/dL)	12.5 ± 1.8	11.8 ± 2.0	12.8 ± 1.6	<0.001
WBC (×10^9^/L)	7.5 ± 2.1	8.2 ± 2.5	7.2 ± 1.9	<0.001
Platelets (×10^9^/L)	250 ± 70	265 ± 80	245 ± 65	0.002

CRP—C-reactive protein; WBC—White blood cells.

**Table 3:** Surgical details.

Surgical Variable	Total (n = 688)	Complications (n = 196)	No Complications (n = 492)	*p*-Value
Laparoscopic Surgery (%)	312 (45.3%)	68 (34.7%)	244 (49.6%)	<0.001
Operative Time (minutes)	180 ± 45	195 ± 50	175 ± 40	<0.001
Blood Loss > 500 mL (%)	128 (18.6%)	76 (38.8%)	52 (10.6%)	<0.001
Intraoperative Complications (%)	60 (8.7%)	36 (18.4%)	24 (4.9%)	<0.001
Type of Resection (%)				0.004
- Right Hemicolectomy	206 (29.9%)	52 (26.5%)	154 (31.3%)	0.196
- Left Hemicolectomy	142 (20.6%)	38 (19.4%)	104 (21.1%)	0.642
- Sigmoidectomy	220 (32.0%)	68 (34.7%)	152 (30.9%)	0.351
- Low Anterior Resection	120 (17.4%)	38 (19.4%)	82 (16.7%)	0.399

**Table 4:** Postoperative outcomes.

Outcome Variable	Total (n = 688)	Complications (n = 196)	No Complications (n = 492)	*p*-Value
Length of Hospital Stay (Days)	10 ± 4	14 ± 5	8 ± 3	<0.001
Reoperation Rate (%)	40 (5.8%)	32 (16.3%)	8 (1.6%)	<0.001
30-day Mortality (%)	36 (5.2%)	28 (14.3%)	8 (1.6%)	<0.001
Clavien–Dindo Classification (%)			−4.3 ± 2.1	<0.001
Grade I–II	112 (16.3%)	112 (57.1%)	0	-
Grade III–IV	84 (12.2%)	84 (42.9%)	0	-
Bowel Function Recovery (Days)	5.7 ± 1.8	7.4 ± 2.2	4.9 ± 1.3	<0.001
Continence at 30 Days (%)	625 (90.8%)	160 (81.6%)	465 (94.5%)	<0.001

**Table 5:** Subgroup analyses.

Subgroup/Sensitivity Analysis	Group 1: Elevated CRP Levels	Group 2: Normal CRP Levels	*p*-Value
Age < 65 years	Complications: 43.6% (34.2–51.7)	Complications: 18.7% (13.3–22.8)	0.003
Age ≥ 65 years	Complications: 67.9% (60.4–73.9)	Complications: 31.2% (26.9–35.8)	<0.001
Laparoscopic surgery	Complications: 28.4% (22.1–35.9)	Complications: 15.8% (11.3–20.7)	0.008
Open surgery	Complications: 62.3% (55.6–69.8)	Complications: 34.1% (29.2–40.3)	<0.001
BMI ≥ 25 kg/m^2^	Complications: 39.7% (33.2–46.9)	Complications: 21.9% (17.5–27.6)	0.004
BMI < 25 kg/m^2^	Complications: 54.6% (46.3–62.4)	Complications: 26.8% (21.7–32.9)	0.002

CI—Confidence interval; OR—Odds ratio; BMI—Body mass index; CRP—C-reactive protein.

**Table 6:** Univariate analysis of factors associated with postoperative complications.

Variable	OR	95% CI	*p*-Value
Age (>65 years)	1.8	1.3–2.4	<0.001
BMI (<18.5 kg/m^2^)	1.5	1.1–2.0	0.012
Smoking	1.5	1.1–2.1	0.015
Alcohol Consumption	1.5	1.1–2.2	0.018
Diabetes Mellitus	1.8	1.3–2.5	<0.001
Hypertension	1.4	1.0–1.9	0.036
ASA Score (≥3)	3.5	2.5–4.9	<0.001
Elevated CRP	2.3	1.7–3.1	<0.001
Low Albumin	2	1.5–2.7	<0.001
Hemoglobin (<10 g/dL)	1.9	1.4–2.6	<0.001
WBC (>10 × 10^9^/L)	1.7	1.2–2.3	0.003
Laparoscopic Surgery (No)	1.9	1.4–2.5	<0.001
Operative Time (>180 min)	1.6	1.2–2.2	0.002
Blood Loss (>500 mL)	5.3	3.6–7.8	<0.001
Intraoperative Complications	4.4	2.6–7.3	<0.001

CI—Confidence interval; OR—Odds ratio.

**Table 7:** Multivariate logistic regression analysis of factors associated with postoperative complications.

Variable	Adjusted OR	95% CI	*p*-Value
Age (>65 years)	1.5	1.1–2.1	0.021
ASA Score (≥3)	2.8	1.9–4.1	<0.001
Elevated CRP	2.1	1.5–2.9	<0.001
Low Albumin	1.8	1.3–2.5	<0.001
Blood Loss (>500 mL)	2.4	1.7–3.4	<0.001
Intraoperative Complications	2.9	1.6–5.2	<0.001

## Data Availability

Data available on request.
